# Clinical Outcomes of Vancomycin Powder for Surgical Site Prophylaxis in Orthopedic Surgeries: A Comparative Study

**DOI:** 10.7759/cureus.87151

**Published:** 2025-07-02

**Authors:** Ahmed S Ibrahim, Ahmed M Abdelhai, Awatif Mahmoud, Mohamed Alghazali, Eltayeb Hamza, Mohamed Hajalhasan, Mohammed Mohammed, Ahmed Abbas

**Affiliations:** 1 Trauma and Orthopedics, Nile University, Khartoum, SDN; 2 Trauma and Orthopedics, Royal Care International Hospital, Khartoum, SDN; 3 Trauma and Orthopedics, University of Khartoum, Khartoum, SDN; 4 Anesthesiology, University of Medical Sciences and Technology, Khartoum, SDN; 5 Trauma and Orthopedics, Ibrahim Malik Teaching Hospital, Khartoum, SDN; 6 Trauma and Orthopedics, Sudan International University, Khartoum, SDN; 7 Trauma and Orthopedics, National Ribat University, Khartoum, SDN; 8 Trauma and Orthopedics, University of Gezira, Wad Madani, SDN

**Keywords:** antibiotics, orthopedic surgery, prophylaxis, surgical site injection, vancomycin powder

## Abstract

Background: Surgical site infections (SSIs) remain a significant challenge in orthopedic surgery, despite existing methods for lowering SSI rates, contributing to increased morbidity, mortality, and healthcare costs. The search for more effective prophylaxis strategies is a major area of research to reduce postoperative morbidity and mortality. Topical application of vancomycin powder (VP) has been suggested as an adjunctive measure for reducing SSIs.

Objective: To evaluate the effectiveness of intraoperative topical VP in preventing SSIs in orthopedic surgeries.

Methods: A comparative hospital-based observational study was conducted at Future Hospital, Khartoum, from September 2021 to October 2022. A total of 300 patients undergoing orthopedic surgery were enrolled and divided into two equal groups: a VP group (n=150) receiving intra-wound VP and a control group (n=150) without VP. Data on postoperative SSI and other variables were collected from hospital records and analyzed using IBM SPSS Statistics for Windows, Version 26 (Released 2018; IBM Corp., Armonk, New York, United States).

Results: A total of 300 patients were included. The mean patient age was 42.4±19.5 years, and 205 patients (68.3%) were male. There were no significant differences between observed cases and control groups in age, gender, occupation, diagnosis, and type of operations. Fractures were the most common indication for surgery (158 patients, 52.7%), and decompression with fixation was the most frequent procedure (143 patients, 47.7%). Patient age was significantly associated with SSIs (p=0.009), with older patients showing higher rates. The SSI rate was significantly lower in the topical VP group (four patients, 2.7%) compared to the control group (11 patients, 7.3%) (P=0.042). No vancomycin-related adverse reactions were observed.

Conclusion: The intraoperative application of VP significantly reduced the incidence of SSIs in orthopedic surgeries. Routine use of VP should be considered, especially in high-risk patients.

## Introduction

Surgical site infection (SSI) is a common and serious nosocomial infection, ranking as the third most frequently reported [1]. SSIs impose a substantial burden on patients and healthcare systems, leading to increased morbidity, prolonged hospital stays, higher treatment costs, and potentially increased mortality [2]. In orthopedic surgery, SSIs can be particularly devastating due to the involvement of bone, joints, and implanted materials, often making eradication difficult and detection delayed [3]. For example, the overall SSI rate in the United States between 2006 and 2008 was reported at 2%, with the highest incidence in open fracture surgeries and the lowest in elective joint arthroplasty surgeries [3]. While the causes of SSIs are multifactorial, recognized risk factors include the duration and complexity of the surgery, as well as the length of hospital stay and patient comorbidities [4].

Various measures are employed to reduce SSI rates, including meticulous hygiene, the use of appropriate surgical clothing, optimized operating room airflow, and the administration of prophylactic antibiotics [3]. However, the challenge of SSI persists, especially with the rise of antibiotic-resistant organisms like methicillin-resistant *Staphylococcus aureus* (MRSA). The presence of microorganisms in the surgical site postoperatively is associated with an increased risk of SSI. Consequently, ongoing research is being conducted into more effective prophylactic strategies [5]. For example, studies showed that intraoperative irrigation with solutions like normal saline or antiseptics (e.g., povidone-iodine) revealed good outcomes by decreasing the incidence of SSI [6].

The local administration of topical powdered antibiotics, first explored in the 1960s for abdominal surgeries [7], offers an attractive method to deliver high antibiotic concentrations directly to the surgical site with minimal systemic exposure and fewer adverse effects [8]. In addition, local administration of topical powdered antibiotics can be useful in areas with compromised vascularity [9]. Vancomycin, a glycopeptide antibiotic effective against Gram-positive bacteria, including MRSA, inhibits cell wall synthesis [10]. A typical dose of one to two grams applied directly into the wound has been recommended, particularly for high-risk patients [11].

Previous studies investigated the potential role of VP in reducing SSI. For example, Qadir et al. reported a significant reduction in infection rates in high-risk fractures with VP [12]. Similarly, Dial et al. found a lower deep infection rate in primary total hip arthroplasty patients receiving VP [13]. Conversely, Lemans JV et al. [14] found a reduction in superficial SSIs but no significant difference in deep SSIs in spine surgery with VP, while randomized trials by Tubaki VR et al. [15] and Mirzashahi B et al. [16] reported no benefit. These differing results highlight the need for context-specific evidence and further research.

Despite its potential benefits, the routine use of vancomycin powder (VP) for SSI prophylaxis in orthopedic surgery, particularly in regions like Sudan, lacks strong local evidence-based support. This study aimed to assess the impact of using VP in the surgical site as prophylaxis for SSI incidence in orthopedic surgeries and to assess any associated complications related to VP application. Given the logistical considerations and practical challenges of randomizing patients in this context, a comparative observational design was chosen to evaluate the effectiveness of intraoperative VP in orthopedic surgeries.

## Materials and methods

Study settings and participants

This was an observational, comparative, hospital-based study conducted at Future Hospital in Khartoum, Sudan, between September 2021 and October 2022. Future Hospital is considered one of the important specialized orthopedic centers in the state, serving a wide urban and rural population, equipped with three operating rooms, four ICU beds, and an advanced sterilization center.

A total of 300 patients undergoing orthopedic operations were included. Patients were selected based on hospital records. Inclusion criteria were patients aged <85 years undergoing clean orthopedic surgeries who had consented for their data to be used for research. Exclusion criteria included patients with intraoperative complications (e.g., significant intraoperative bleeding, iatrogenic fracture), open fractures, pre-existing infected wounds, or significant comorbidities (e.g., uncontrolled diabetes mellitus, hypertension, or renal diseases).

In this non-randomized comparative study, patients were retrospectively grouped for analysis based on the treatment administered during their surgery. The participants were divided into two study groups. The VP group (n=150) received intra-wound VP (1-2 g) after saline irrigation and before wound closure, while the control group (n=150) received standard care without application of VP. Antiseptic measures were maintained for all participants, and surgeries were conducted in a laminar airflow theater. All patients received a weight-dependent intravenous dose of cefuroxime during the induction of anesthesia. All patients received intravenous cefuroxime at induction and continued for 72 hours postoperatively. Wounds were closed in layers using absorbable Vicryl® (Ethicon, Inc., NJ, USA) deep) and Monocryl® (Ethicon, Inc., NJ, USA) (subcuticular to skin) sutures, followed by skin stapler application. Suction drains were inserted depending on the surgery.

Data collection and analysis

The primary independent outcome was the incidence of SSIs. The SSIs were diagnosed based on clinical signs and symptoms, as assessed by the operating surgeons. All patients were observed for one year postoperatively to assess any early or late signs of SSI. Secondary variables included demographics (age, gender, residence, and occupation) and clinical data (indication for surgery, type of surgical procedure, diagnosis, and type of operation). Data were collected from hospital records using a pre-designed questionnaire (Appendix 1).

Descriptive statistics (mean, standard deviation, frequencies, and percentages) were used to summarize the data. The chi-square test was used for categorical variables, and the independent samples t-test was used for continuous variables to compare the two groups. The significance level for all analyses was set at p < 0.05, and all analyses were conducted using IBM SPSS Statistics for Windows, Version 26 (Released 2018; IBM Corp., Armonk, New York, United States).

Ethical considerations

Ethical clearance and approval for conducting this research were obtained from the Sudan Medical Specialization Board Ethical Committee and Educational Development Center (2021-10-001). Permission to conduct this research was obtained from the administrative authority of the Future Hospital. Study data were used for research purposes only. Consents were taken from all participants, and confidentiality was ensured for all the participants.

## Results

Baseline characteristics

A total of 300 patients were analyzed: 150 in the VP group and 150 in the control group. The mean age was 42.4±19.5 years, with a male-to-female ratio of 2.1:1. The majority of patients were Sudanese (98.7%) and resided in Khartoum state (71.3%). No significant differences were observed between groups regarding baseline characteristics in terms of nationality (P=0.566), residence (P=0.444), gender (P=0.710), or occupation (P=0.10) (Table 1).

**Table 1:** Demographic and clinical characteristics of study participants (VP vs. no VP groups) VP: vancomycin; X^2^: chi-square test

Variable	Category	VP Group (n=150)	No VP Group (n=150)	Total (n=300)	X^2^	P-value
Nationality	Sudan	148 (49.3%)	148 (49.3%)	296 (98.7%)	0.33	0.566
	South Sudan	2 (0.7%)	1 (0.3%)	3 (1.0%)		
	Yemen	0 (0.0%)	1 (0.3%)	1 (0.3%)		
Residence	Khartoum	104 (34.7%)	110 (36.7%)	214 (71.3%)	0.66	0.444
	Outside	46 (15.3%)	40 (13.3%)	86 (28.7%)		
Gender	Female	49 (16.3%)	46 (15.3%)	95 (31.7%)	0.80	0.710
	Male	101 (33.7%)	104 (34.7%)	205 (68.3%)		
Occupation	Worker	4 (1.3%)	12 (4.0%)	16 (5.3%)	0.25	0.10
	Employee	26 (8.7%)	8 (2.7%)	34 (11.3%)		
	Housewife	33 (11.0%)	23 (7.7%)	56 (18.7%)		
	Freelancer	39 (13.0%)	63 (21.0%)	102 (34.0%)		
	Student	30 (10.0%)	29 (9.7%)	59 (19.7%)		
	Not working	18 (6.0%)	15 (5.0%)	33 (11.0%)		

Clinical characteristics

The most common diagnoses were fractures (52.7%) and spinal canal stenosis (37.7%). The most frequently performed procedures were decompression with fixation (47.7%) and open reduction and internal fixation (34.3%). There were no significant differences between the VP and control groups regarding the indication for surgery (P=0.225) or the type of operation performed (P=0.223). No adverse drug reactions were reported among the VP patient group (Table 2).

**Table 2:** Clinical diagnoses and types of operations AVN: avascular necrosis; ORIF: open reduction with internal fixation; PSF: posterior spinal fusion; VP: vancomycin; X^2^: chi-square test

Variable	Category	VP Group (n=150)	No VP Group (n=150)	Total (n=300)	X^2^	P-value
Indications	Fracture	81 (27.0%)	77 (25.7%)	158 (52.7%)	8.17	0.225
	Canal stenosis	57 (19.0%)	56 (18.7%)	113 (37.7%)		
	Scoliosis	8 (2.7%)	7 (2.3%)	15 (5.0%)		
	Osteoarthritis	0 (0.0%)	2 (0.7%)	2 (0.7%)		
	AVN hip	2 (0.7%)	0 (0.0%)	2 (0.7%)		
	Kyphosis	0 (0.0%)	2 (0.7%)	2 (0.7%)		
	Other diagnoses	2 (0.7%)	6 (2.0%)	8 (2.7%)		
Operations	Arthrodesis	3 (1.0%)	0 (0.0%)	3 (1.0%)	10.63	0.223
	Arthroplasty	8 (2.7%)	7 (2.3%)	15 (5.0%)		
	Biopsy	0 (0.0%)	2 (0.7%)	2 (0.7%)		
	Decompression + fixation	76 (25.3%)	67 (22.3%)	143 (47.7%)		
	Implant removal	6 (2.0%)	5 (1.7%)	11 (3.7%)		
	ORIF	45 (15.0%)	58 (19.3%)	103 (34.3%)		
	Other operations	4 (1.3%)	2 (0.7%)	6 (2.0%)		
	PSF	2 (0.7%)	0 (0.0%)	2 (0.7%)		
	PSF + osteotomy	6 (2.0%)	9 (3.0%)	15 (5.0%)		

Comparative analyses of rates and associated factors with surgical site infections

Postoperative SSI was reported in 15 out of 300 participants (5.0%). The SSI rate was significantly lower in the VP group, with only four patients (2.7%) developing SSI in the VP group, compared to 11 patients (7.3%) in the control group (P=0.042). In addition to VP exposure status, patients' age was significantly associated with SSI. The mean age of patients who developed SSI was 55.33±16.5 years, compared to 41.76±19.5 years for those who did not develop SSI (P=0.009). On the other hand, there was no statistically significant association between SSI and patient gender (P=0.887), indication for surgery (P=0.203), or type of operation (P=0.509) (Table 3).

**Table 3:** Analysis of factors with associated SSI AVN: avascular necrosis; ORIF: open reduction with internal fixation; PSF: posterior spinal fusion; VP: vancomycin; SSI: surgical site infection; X^2^: chi-square test; t: t-test

Variable	Category	Non-SSI (n=285)	SSI (n=15)	Test	P-value
Age (years)	Age (mean ± SD)	41.76 ± 19.5	55.33 ± 16.5	t=2.65	0.009
Application of VP	VP group	146 (97.3%)	4 (2.7%)	X^2^=3.43	0.0427
	No VP group	139 (92.7%)	11 (7.3%)		
Gender	Female	90 (30.0%)	5 (1.7%)	X^2^=0.02	0.887
	Male	195 (65.0%)	10 (3.3%)		
Indications	Fracture	149 (49.7%)	9 (3.0%)	X^2^=8.5	0.203
	Canal stenosis	109 (36.3%)	4 (1.3%)		
	Scoliosis	15 (5.0%)	0 (0.0%)		
	Osteoarthritis	2 (0.7%)	0 (0.0%)		
	AVN hip	2 (0.7%)	0 (0.0%)		
	Kyphosis	2 (0.7%)	0 (0.0%)		
	Other	6 (2.0%)	2 (0.7%)		
Operations	Arthrodesis	3 (1%)	0 (0.0%)	X^2^=7.26	0.509
	Arthroplasty	14 (4.7%)	1 (0.3%)		
	Biopsy	2 (0.7%)	0 (0.0%)		
	Decompression + fixation	136 (45.3%)	7 (2.3%)		
	Excision	9 (3%)	2 (0.7%)		
	ORIF	99 (33%)	4 (1.3%)		
	Other operations	5 (1.7%)	1 (0.3%)		
	PSF	2 (0.7%)	0 (0.0%)		
	PSF + osteotomy	15 (0.5%)	0 (0.0%)		

## Discussion

SSI is a serious complication in orthopedic surgery, often leading to extensive treatment, extended hospitalization, long-term antibiotic medications, and increased healthcare costs [17]. Since *Staphylococcus aureus* is a common causative pathogen of SSI, perioperative strategies to mitigate SSI risk have been used to reduce the incidence of infection and the rising bacterial resistance [18]. Our findings support the use of topical VP as an effective method for reducing SSIs in orthopedic surgeries. The significant reduction in infection rate (2.7% vs. 7.3%) aligns with results from other studies demonstrating protective effects of VP in orthopedic surgeries [12,13]. A meta-analysis by Sadigursky et al. showed a statistically significant protective effect of local VP against SSI in spine surgery (RR 0.59) [19]. 

Local application of VP offers a method to achieve high local antibiotic concentrations with minimal systemic absorption [20]. On the other hand, intravenous vancomycin has limitations, including potential renal toxicity and poor penetration into certain tissues [21]. Importantly, the study results revealed that no adverse effects attributable to VP, such as hypersensitivity reactions or sterile seromas, were observed in our study population. This is a reassuring finding, as rare instances of circulatory collapse [22] or sterile seroma formation [23] have been reported in the literature, although causality in those cases was not definitively established solely to vancomycin. Thus, careful patient selection and monitoring are essential.

Our study also identified a significant association between older age and an increased risk of SSI. However, unlike some studies, we did not find a significant association between SSI and gender, surgical indication, or type of operation. Hill et al. noted differences in risk factor distribution in their cohorts, but covariate analysis did not link these directly to higher infection risk in their specific study [24].

The study has an important clinical implication. The application of vancomycin post-operatively before skin closure is recommended to be adopted in orthopedic surgeries. However, further studies with different concentrations of vancomycin should be implemented in order to give high-quality evidence about the adverse effects and dosage of this antibiotic.

This study has limitations. It was an observational, non-randomized, single-center study, which may limit the generalizability of the findings. No standardized criteria or microbiological confirmation were uniformly applied to diagnose SSI, and the outcome assessment was not blinded. Also, the study population included a heterogeneous group of orthopedic procedures and indications such as trauma and degenerative conditions, which might introduce confounding variables and limit the generalizability of the findings. Unmeasured confounding may still influence the results. Therefore, while our findings offer valuable real-world insights, they should be interpreted with caution. In the future, prospective multicenter randomized controlled trials are needed to validate these findings and to better explore optimal dosing strategies and long-term outcomes.

## Conclusions

The application of intra-wound VP before wound closure significantly reduced the rate of SSI in orthopedic surgeries. No adverse effects related to VP application were observed among patients.

These findings suggest that local VP can be a safe and effective prophylactic measure to reduce SSIs in orthopedic surgery. Further prospective, randomized controlled trials are needed to confirm these benefits and to explore optimal dosing and application in specific orthopedic subspecialties.

## Figures and Tables

**Figure 1:**
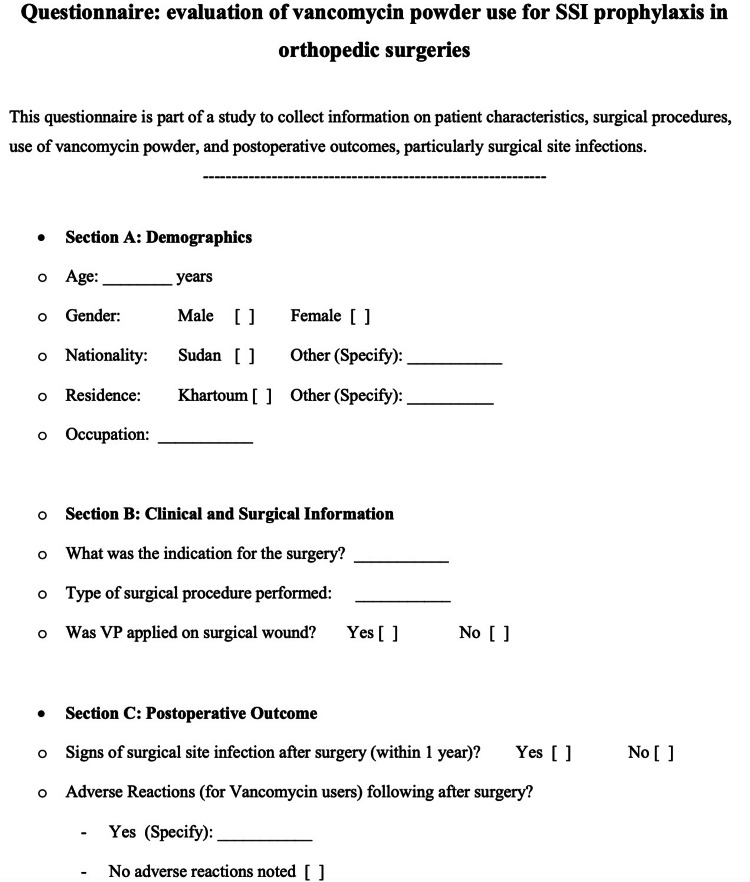
Questionnaire SSI: surgical site infection

## Appendices

Appendix 1
